# S-Nitrosoglutathione Acts as a Small Molecule Modulator of Human Fibrin Clot Architecture

**DOI:** 10.1371/journal.pone.0043660

**Published:** 2012-08-20

**Authors:** Ryon M. Bateman, Christopher G. Ellis, Makoto Suematsu, Keith R. Walley

**Affiliations:** 1 Department of Biochemistry, School of Medicine, Keio University, Tokyo, Japan; 2 Heart Lung Institute, University of British Columbia, Vancouver, Canada; 3 Department of Medical Biophysics, University of Western Ontario, London, Canada; Leiden University Medical Center, The Netherlands

## Abstract

**Background:**

Altered fibrin clot architecture is increasingly associated with cardiovascular diseases; yet, little is known about how fibrin networks are affected by small molecules that alter fibrinogen structure. Based on previous evidence that S-nitrosoglutathione (GSNO) alters fibrinogen secondary structure and fibrin polymerization kinetics, we hypothesized that GSNO would alter fibrin microstructure.

**Methodology/Principal Findings:**

Accordingly, we treated human platelet-poor plasma with GSNO (0.01–3.75 mM) and imaged thrombin induced fibrin networks using multiphoton microscopy. Using custom designed computer software, we analyzed fibrin microstructure for changes in structural features including fiber density, diameter, branch point density, crossing fibers and void area. We report for the first time that GSNO dose-dependently decreased fibrin density until complete network inhibition was achieved. At low dose GSNO, fiber diameter increased 25%, maintaining clot void volume at approximately 70%. However, at high dose GSNO, abnormal irregularly shaped fibrin clusters with high fluorescence intensity cores were detected and clot void volume increased dramatically. Notwithstanding fibrin clusters, the clot remained stable, as fiber branching was insensitive to GSNO and there was no evidence of fiber motion within the network. Moreover, at the highest GSNO dose tested, we observed for the first time, that GSNO induced formation of fibrin agglomerates.

**Conclusions/Significance:**

Taken together, low dose GSNO modulated fibrin microstructure generating coarse fibrin networks with thicker fibers; however, higher doses of GSNO induced abnormal fibrin structures and fibrin agglomerates. Since GSNO maintained clot void volume, while altering fiber diameter it suggests that GSNO may modulate the remodeling or inhibition of fibrin networks over an optimal concentration range.

## Introduction

Changes in fibrin clot architecture are increasingly recognized as an important risk factor for cardiovascular disease and thrombotic complications [1]–[5]. Fibrin clots form when thrombin catalyzes the conversion of fibrinogen to fibrin monomers that form double stranded protofibrils that elongate, branch (diverge and converge to form predominantly trifunctional branch junctions [6]–[8]), and laterally associate to form a three-dimensional fibrin network, visible at the gel point. Concomitantly, thrombin activated FXIII stabilizes and strengthens the fibrin network by cross-linking protofibrils and fibers [9]. The composition of a network’s structural elements, including fiber density, diameter and branch point density [8] are determined by the clotting conditions, including fibrinogen, thrombin and calcium concentrations, FXIIIa activity, pH, ionic strength, temperature, whether fibrin is formed in plasma and by changes in fibrinogen structure [8], [10]–[16].

S-nitrosoglutathione (GSNO) is a low molecular weight member of a class of molecules referred to as S-nitrosothiols that have emerged as important biological signaling molecules and important mediators of liver damage and animal survival during bacterial infection [17]. In blood, S-nitrosothiols/GSNO play an essential role in vascular homeostasis [17]–[19] and have relative plasma concentrations of 7 uM/100–300 nM, respectively [18], [20], which can increase 10–30 fold in pneumonia or the absence of GSNO reductase [17], [18], [21]. Additionally, GSNO modulates hemostasis by inhibiting platelet activation and aggregation, and decreasing fibrinogen binding to platelets [22]–[25]. GSNO has been used clinically as an antithrombotic agent [26], [27] and is also being incorporated into drug-eluting stents to prevent restenosis [28].

GSNO has been reported to bind to fibrinogen α-chain c-termini, altering fibrinogen secondary structure and fibrin polymerization kinetics [29], [30] and inhibit factor XIIIa activity and fiber cross-linking in a dose-dependent manner [31]. However, the effect of GSNO on fibrin clot architecture is unknown. To test the hypothesis that GSNO alters fibrin network microstructure, we used multiphoton microscopy to image human fibrin clots in their native state at 37°C and developed computer software to analyze clot structural elements. We found that over a four-fold concentration range GSNO shifted clot structure towards less dense coarse clots with thicker fibers, ultimately inhibiting clot formation. Additionally, at progressively higher GSNO concentrations, first abnormal fibrin clusters developed within fibrin networks and ultimately fibrin networks were replaced by fibrin agglomerates. To the best of our knowledge, this is the first report of GSNO inducing fibrin particle formation. Taken together, GSNO altered human fibrin clot formation in such a way that network structures went beyond the bounds of the classically defined clot extremes of “fine” and “course” clots [14].

## Methods

Human plasma was obtained from the Canadian Blood Services blood bank for research purposes in accordance with research policies at the University of British Columbia. Data were analyzed anonymously. Plasma was collected from three healthy volunteers in acid citrate dextrose tubes and pooled as previously described [9], [32]. All experiments were performed in triplicate.

### Fibrin Polymerization of Human Platelet-poor Plasma (PPP) Treated with S-nitrosoglutathione (GSNO)

Platelet-poor plasma (PPP) was prepared by centrifuging plasma samples at 3000rpm for 20 minutes [1]. Aliquots were frozen at −80°C and thawed once on the day of each experiment. Fibrin polymerization reactions were carried out in 27 µL final volumes as follows: 21 µL PPP was mixed with 2 µL of S-nitrosoglutathione (GSNO, AG Scientific, Inc, San Diego, CA, final concentrations ranged from 10 µM–3.75 mM) or 2 µL PBS (phosphate buffered saline, ph 7.4, Sigma, St.Louis, MO) and incubated for 20 minutes in a closed tube placed in a 37°C water bath. The sample was then supplemented with 1 µL CaCl_2_ (Sigma, St.Louis, MO, final concentration 18 mM) and 1 µL Oregon Green labeled fibrinogen (5% vol/vol) to provide fluorescent imaging contrast. Lastly, the sample was rapidly mixed with 2 µL human thrombin (Chrono-log, Havertown, PA, final PPP concentration 1 U/ml). In control experiments, tissue plasminogen activator (tPA)/thrombin mixtures (ratios of 1/20 and 1/100) (Molecular Innovations Inc, Peary Court, MI) were used to verify that Oregon Green labeled fibrinogen did not interfere with normal clot formation and fibrinolysis. Once fibrin polymerization had been initiated, the PPP sample was quickly spotted on a cover slip pretreated with albumin to reduce surface effects and incubated (37°C) in the dark for 20 minutes. Since fibrin polymerization occurred rapidly under these conditions, we carefully applied the sample immediately after the onset of polymerization and let the fibrin clot develop in the standing platelet-poor plasma droplet. Samples reached their gel point in less than 5 minutes, with the exception of the highest GSNO concentration tested which failed to form a fibrin clot.

### Multiphoton Microscopy (MPM)

Multiphoton microscopy is a relatively new imaging technology made possible by the development of femtosecond pulse lasers. In contrast to fluorescent confocal microscopy or spinning-disc microscopy where a single photon is used to excite a given fluorophore, in multiphoton microscopy (MPM), two photons of longer wavelength are used to simultaneously excite a sample fluorophore [33]. The advantage of MPM is that longer wavelengths allow for deeper sample penetration of both tissues [34] and fluids with less light scattering, less photobleaching and loss of fluorescent signal, as may occur with confocal microscopy. This occurs because multiphoton excitation is non-linear and limited to a small femtoliter focal volume at the focal point of the laser beam; whereas, one photon imaging creates a large cone of excitation within the sample. Nevertheless, multiphoton imaging can require high powers to excite imaging targets and may induce photobleaching. Accordingly, caution was taken in these experiments to limit sample exposure to laser energy in order to maintain fluorescent fiber structural integrity.

### Fibrin Clot Image Acquisition Using Multiphoton Microscopy

Images were acquired twenty minutes after the onset of polymerization, and at least 15 minutes beyond the sample gel point. There was no evidence of fiber movement at the time of imaging indicating the clot network was stable. 900 nm fs pulses with a FWHM of 14 nm (Ti-Sapphire laser, Spectra Physics, Santa Clara, CA) were focused through a 63×/1.2 NA water immersion objective mounted on an inverted Leica AOBS SP2 microscope system, (Leica Microsystems GmbH, Wetzlar, Germany). Fluorescent images (1024×1024 pixels, at 2 and 4× optical zoom, 8 bit image depth) were acquired using 400 Hz/3frame averages and an emission bandwidth of 500–650 nm. We imaged from the surface of the clot to a height of 100 microns and discovered that Nyquist sampling (∼20 minute scan time) introduced sufficient energy to cause sample evaporation. To eliminate this problem and avoid introducing structural artifacts, yet still obtain images through the clot, we limited optical sectioning to 10 micron intervals to a height of 100 microns within the clot. Using this imaging protocol, we found no evidence of either sample evaporation or photobleaching.

**Figure 1:**
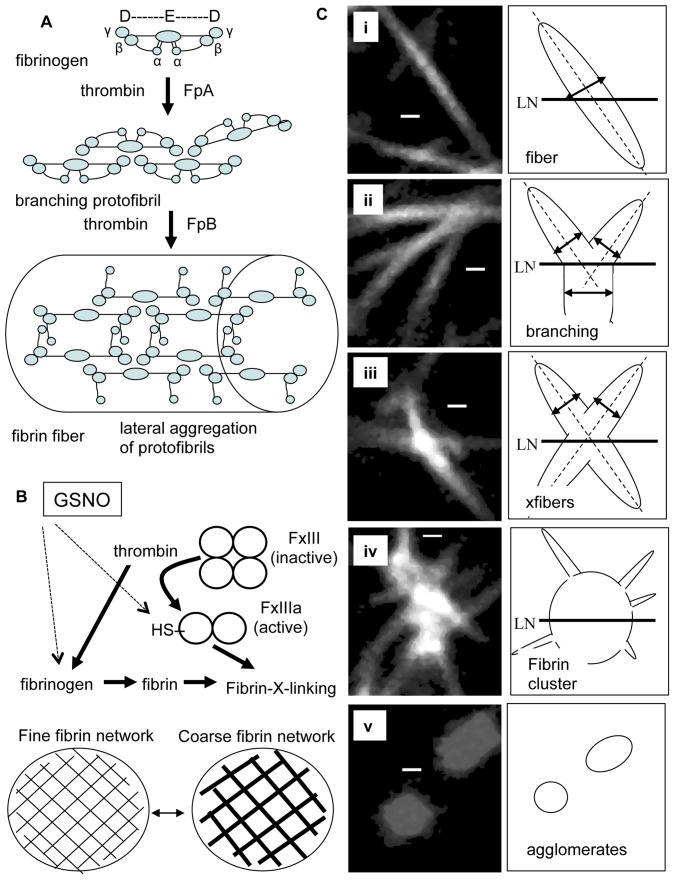
Fibrin polymerization, GSNO targets and fibrin structural elements imaged by multiphoton microscopy. Panel A shows a schematic of fiber formation. Fibrinogen is a bisymmetrical molecule consisting of three pairs of polypeptide chains (Aα, Bβ, γ)_2_ held together by 29 disulfide bonds. It has a trinodal structure, with central E domain containing N-terminal regions of all six chains connected, by two coiled coils, to two end D domains containing the carboxy-terminal ends of the Bβ and γ chains. The carboxy-terminal portion of the Aα chain folds back from the D domain towards the E domain [8], [48], [49]. Thrombin catalyzes the conversion of fibrinogen to fibrin by cleaving fibrinopeptides A,B from the central E domain. Fibrin monomers self assemble, via complementary E and D domain interactions, forming double stranded half-staggered protofibrils, that branch, elongate, and laterally associate, via released C-terminal α-domains, to form fibrin fibers that constitute the fibrin network [48], [49]. S-nitrosglutathione (GSNO), a low molecular weight endogenous s-nitrosothiol present in blood, targets both fibrinogen altering its secondary structure [29] and the exposed thiol group (R-SH) on factor XIIIa [31] which stabilizes fibrin networks by cross-linking fibrin, panel B. GSNO has no effect on thrombin activity [29]. (solid arrows indicate thrombin targets, while dashed lines indicate GSNO targets.) Clotting conditions control fibrin clot architecture [8] between extreme forms of a fine (thin) fiber and dense network or a thick fiber and coarse (sparse) network, panel B [14]. Panel C shows multiphoton images of fibrin network structural elements, including fibrin fibers (i), fiber branch junctions (ii), crossing fibers (iii), fibrin clusters (iv) and fibrin agglomerates (v). Fibrinogen schematic adapted from Undas et al. [50] Scale bars are 700 nm.

### Image Processing and Quantitative Analysis of Human Fibrin Clot Structures

Images of fibrin networks revealed complex structures that were difficult to analyze by visual inspection. To facilitate rapid quantitative analysis of fibrin clot structural elements, we developed computer software using the MATLAB computing environment (MathWorks, Natick, MA) which allowed us to visualize fibrin structures using intensity and binary images, contour maps and surface plots of fiber intensity. We anticipate that improved imaging methods will enhance quantification of native fibrin networks and will help our understanding of the relationships between endogenous and exogenous factors and fibrin clot architecture. All digital images were subjected to the same background subtraction, normalization and thresholding image processing steps. The starting point for network analysis was the identification of individual fibers and the determination of fibrin fiber density. This was accomplished by sampling images along equally spaced test lines, as shown in Figure S1. Since fiber orientation within a fibrin network is random, the sampling procedure is effectively a random sample of fibrin fibers. The software detected and numbered each fiber on the basis of its fluorescent signal, marked its location and stored its fluorescence intensity and image coordinates and mapped fiber position back into the original image. The computer program is interactive, allowing the user to inspect all identified fibers (Figure S1, panel C) and edit either false positives or false negatives. Since fiber diameter is directly related to lateral aggregation of fluorescently labeled fibrin monomers [9], the program next determined the local maximum fiber intensity and measured the fiber diameter at this location, see Figure S1 for description of fiber measurements. Having established fiber location, calculated fiber density (fibers/100 um) and determined fiber diameter (nm), the user then interactively assessed each identified fiber in the image for any associated trifunctional branch junctions [6], [7] (xbranching) or crossing fibers (xfibers), using contour maps and surface plots at local regions of interest. Note, crossing fibers (xfibers) are distinguished from trifunctional junctions (xbranches) by local regions of high fluorescence intensity indicating that two or more fibers are crossing or in contact at a single point in space, as shown in Figure 1, panels Cii and Ciii.

Figure 1 summarizes fibrin fiber formation, known GSNO coagulation targets (fibrinogen and FXIIIa) and the fibrin network structural elements evaluated in this study. At the end of image analysis, the computer software generated a clot “read out” image listing key fibrin parameters. including nfibers, fiber density (fibers/100 um), fiber intensity (au), fiber diameter (nm), clot void area (%), fiber branching (xbranching/fiber), crossing fibers (xfibers/fiber), fibrin cluster (FbgCluster/mm^2^) and fibrin agglomerate (AG/mm^2^) densities. The program also assessed the distribution of fiber diameters and plotted fiber diameter against fiber intensity, Figure S2, to test the theoretical assumption that fiber diameter was directly related to fiber intensity [9].

**Figure 2:**
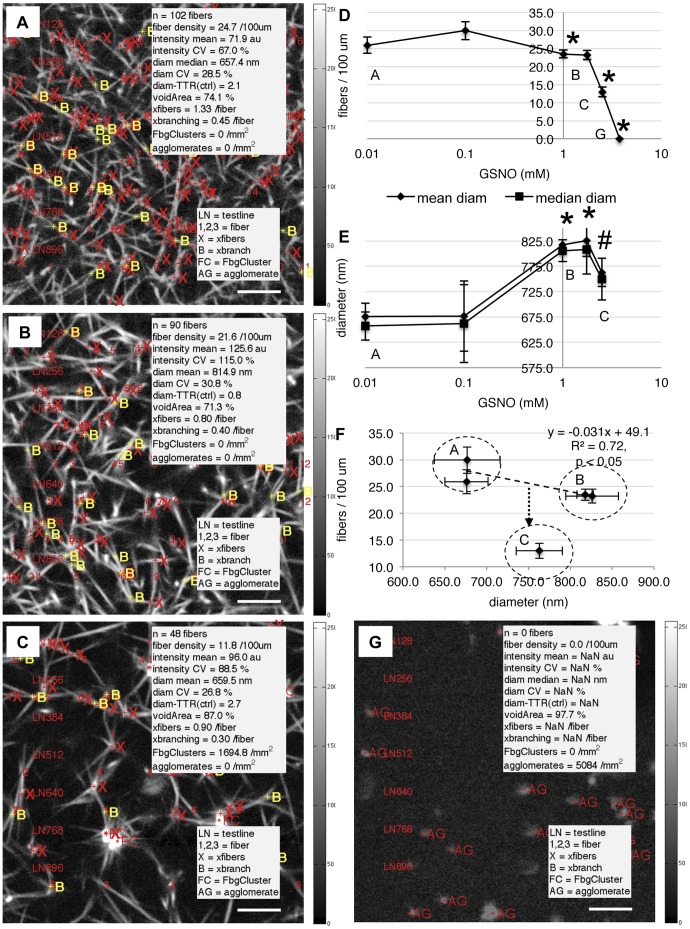
GSNO alters human fibrin fiber density and fiber diameter. Platelet-poor plasma was incubated with GSNO as described in methods. Images of native fibrin clots were acquired using multiphoton microscopy and analyzed using custom designed computer software. Panels A,B,C,G show fibrin clot “read out” images at 0,1,2.5,3.75 mM GSNO, respectively. GSNO decreased fibrin fiber density, panel D, but increased fiber diameter to a maximum at 1 mM GSNO, panel E. Panel F plots fibrin density against fiber diameter. Panel C contained abnormal fibrin clusters with numerous thin diameter fibers protruding from a high intensity core. They decreased the average fiber diameter and shifted the expected density-diameter relationship panel F, point C (2.5 mM GSNO). Fibrin agglomerates were detected at the highest GSNO concentration tested, panel G. Fibrin clot parameters displayed on clot “read out” images: n (number of fibers), fiber density (fibers/100 um), fiber intensity (au, mean and CV), fiber diameter (nm, mean or median and CV), fiber thin-thick ratio vs control (diam-TTR (ctrl)), void area (%), crossing fibers (xfibers/fiber), trifunctional junctions (xbranching/fiber), fibrinogen clusters (FbgClusters/mm^2^) and fibrin agglomerates (AG/mm^2^). *p<0.05 vs control. Area is 59×59 um^2^. Colour bars are image intensity. Scale bars are 8.5 um.

### Statistics

P values <0.05 were considered to be statistically significant. Linear regression was used to evaluate the correlation between fibrin density and fiber diameter. One-way ANOVA was used to test for GSNO dependent differences in fibrin network structural parameters and Dunn’s test was used to test for multiple comparisons within a group. (Sigma Stat 3.5, Systat Software, Inc. Richmond, Ca). Normality plot and maximum likelihood estimation (MLE) MATLAB functions (MathWorks, Natick, MA) were used to statistically analyze and fit diameter data, respectively.

**Figure 3:**
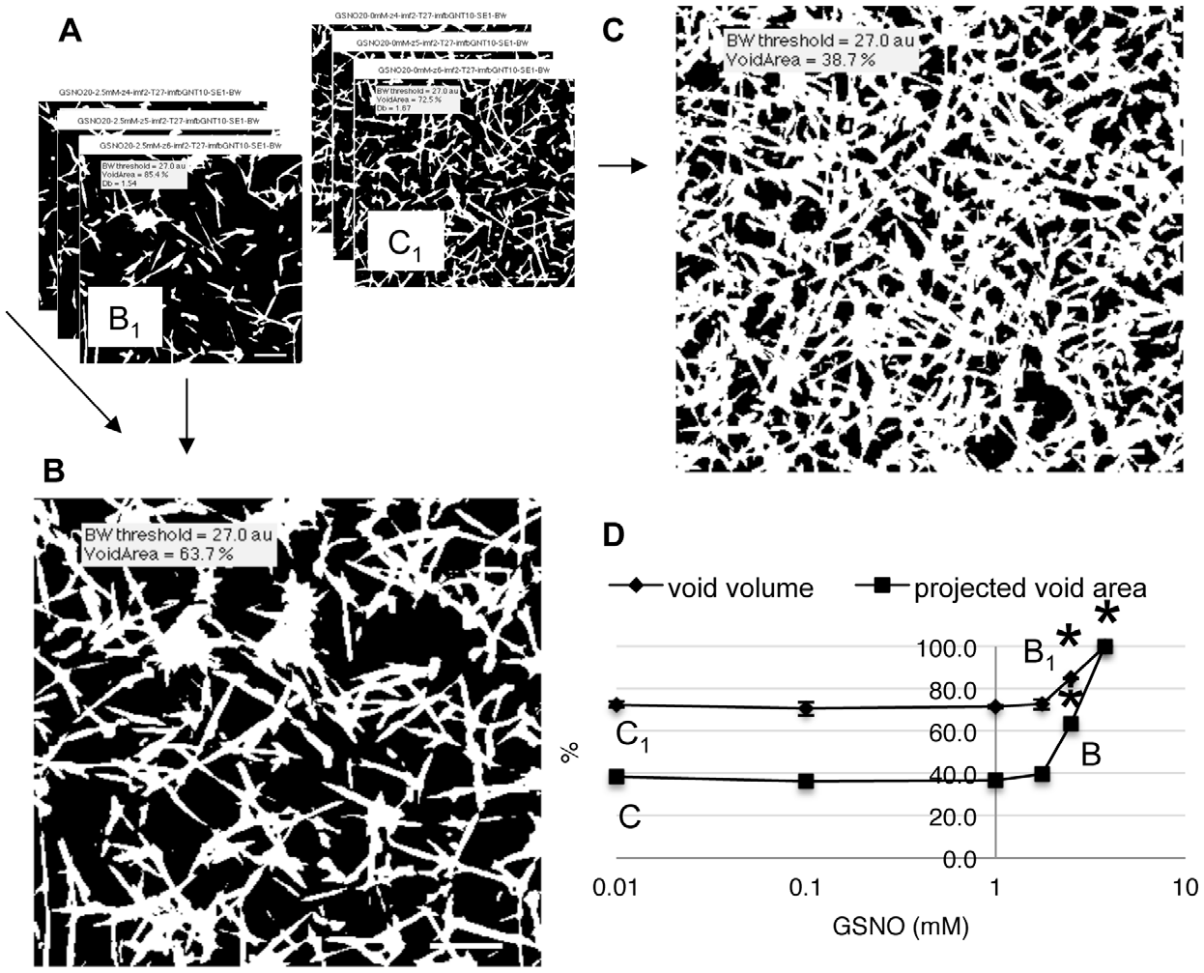
GSNO increases human fibrin clot void volume. Human fibrin clots were prepared and imaged as described in methods. 2D multiphoton images contain spatial information that can be used to quantify clot void volume and projected clot void area from a stack of three images. The calculation uses binary images of clots acquired at 40,50,60 microns above the clot surface, where white pixels are fibers and black pixels are empty space. Panel A shows 3 image stacks for control (C_1_,C_2_,C_3_) and 2.5 mM GSNO (B_1_,B_2_,B_3_), resulting in projected image C and B, respectively. Both clot void volume and projected clot area were insensitive to GSNO at low concentrations, but increased once GSNO exceeded 1.7 mM, panel D. At 3.75 mM GSNO, only fibrin agglomerates were present in plasma, rendering clot void volume effectively 100%, panel D. *p<0.05, compared to control.Scale bars are 8.5 um.

## Results

### GSNO Effects Fiber Density and Fiber Diameter

The first step in computer analysis of fibrin clot networks was the identification of fibrin fibers. Computer analysis was found to have a 98.9% positive predictive value (PPV), where the PPV (%)  =  true positive fiber/(true positive fiber + false positive fiber) *100. Fibers within the fibrin network show a random orientation and we exploited this geometry by horizontally sampling the clot at regular intervals using LN test lines. The corresponding intensity profiles, shown in Figure S1 were then used to identify and number individual fibers. Their respective positions were then mapped back into the original clot image and displayed in fibrin clot “read out” images, as shown in Figure 2, panels A,B,C,G. When human platelet-poor plasma was incubated with increasing concentrations of GSNO, we found fiber density remained relatively unchanged (between 26.4±2.5 and 29.9±2.5 fibers/100 µm) over an order of magnitude (0.01–0.1 mM GSNO), followed by a sharp 53.2% decrease in fiber density as GSNO increased to 2.5 mM, and subsequently to zero, as fibrin formation was completely inhibited, Figure 2D.

**Figure 4:**
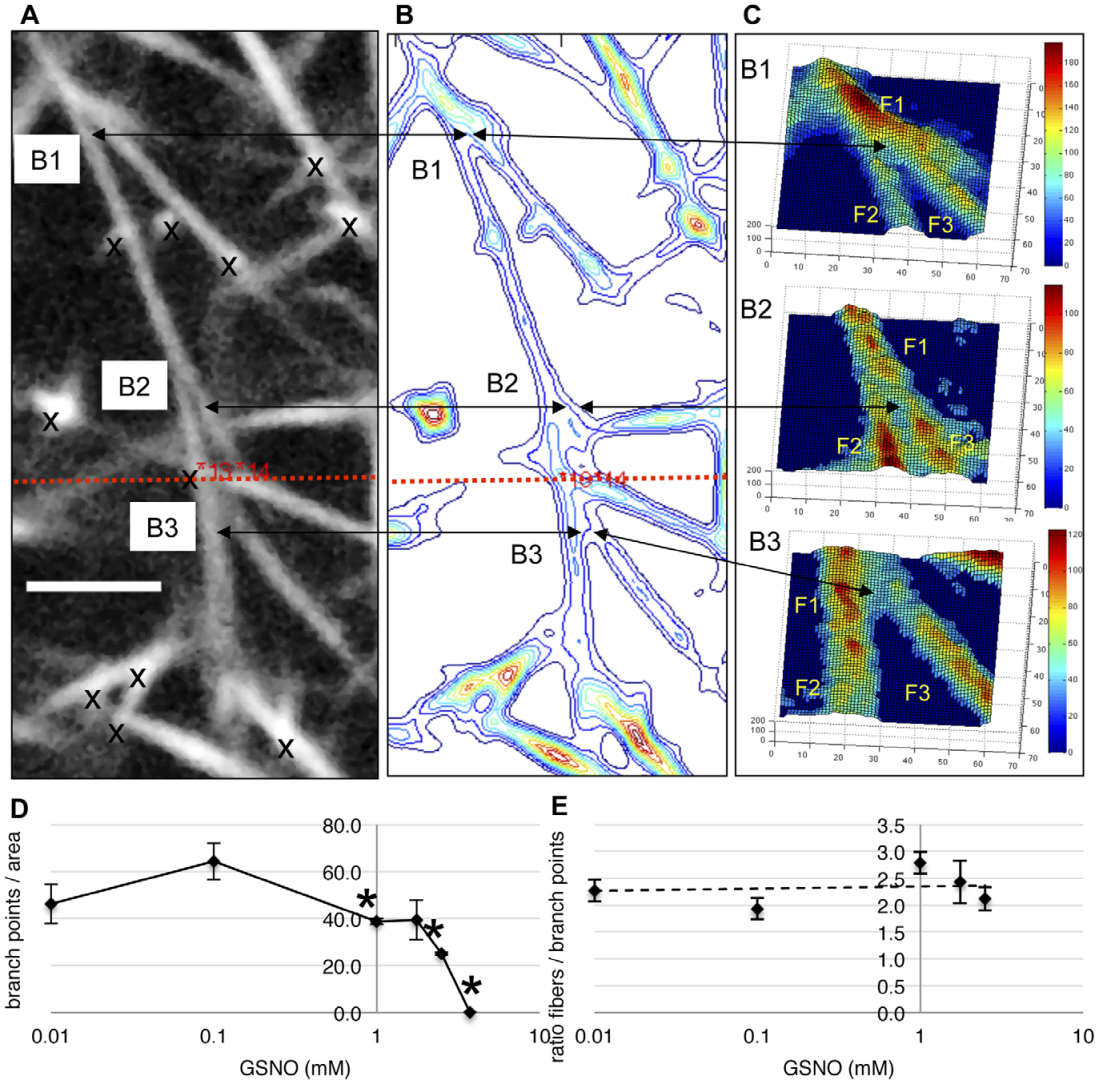
GSNO reduces fibrin branchpoint density, but does not alter the fiber density/branchpoint ratio. Images of human fibrin clots in their native state were acquired using multiphoton microscopy and analyzed using custom designed computer software, as described in methods. Panels A,B,C show a multiphoton image with three branch points (B1,B2,B3) and their corresponding contour map and surface plots, respectively. Branch points are readily identifiable in surface plots, where three fibers intersect at the branch junction and the width of one fiber is approximately equal to the sum of the other fibers (F1 = F2+F3). As GSNO concentration increased, branch junctions per area decreased, panel D. However, the ratio of fiber density to branch point density was insensitive to GSNO concentration, panel E. *p<0.05, compared to control. Area is 59×59 um^2^. Colour bars are image intensity. Scale bar is 4.25 um.

The next step in clot analysis was to measure the diameter of all fibers previously identified in the density analysis. Diameter data was then automatically plotted as a histogram, subjected to a normality statistical test and fit to either a normal or bimodal distribution, as shown in Figure S2. We found that as GSNO concentration increased, mean fiber diameter also increased from 677.4±31.6 nm to a maximum of 822±8.6 nm at 1 mM GSNO, followed by a gradual decrease to 754.3±29.8 nm at 2.5 mM GSNO before fibrin fiber formation was completely inhibited, Figure 2E. Visual inspection of the latter clot images revealed the decrease in mean diameter was associated with the presence of abnormal fibrin cluster structures which had a number of thin fibers protruding from their dense core, Figure 2C. Given the inverse relationship between fiber density and fiber diameter, that is as fiber density decreases fiber diameter increases, plotting fiber density against fiber diameter should yield a straight line. However, fibers formed at 2.5 mM GSNO did not fit on a best fit line between the density-diameter values at lower GSNO concentrations, Figure 2F. Thus the expected fiber density-diameter relationship had been altered by high dose GSNO.

**Figure 5:**
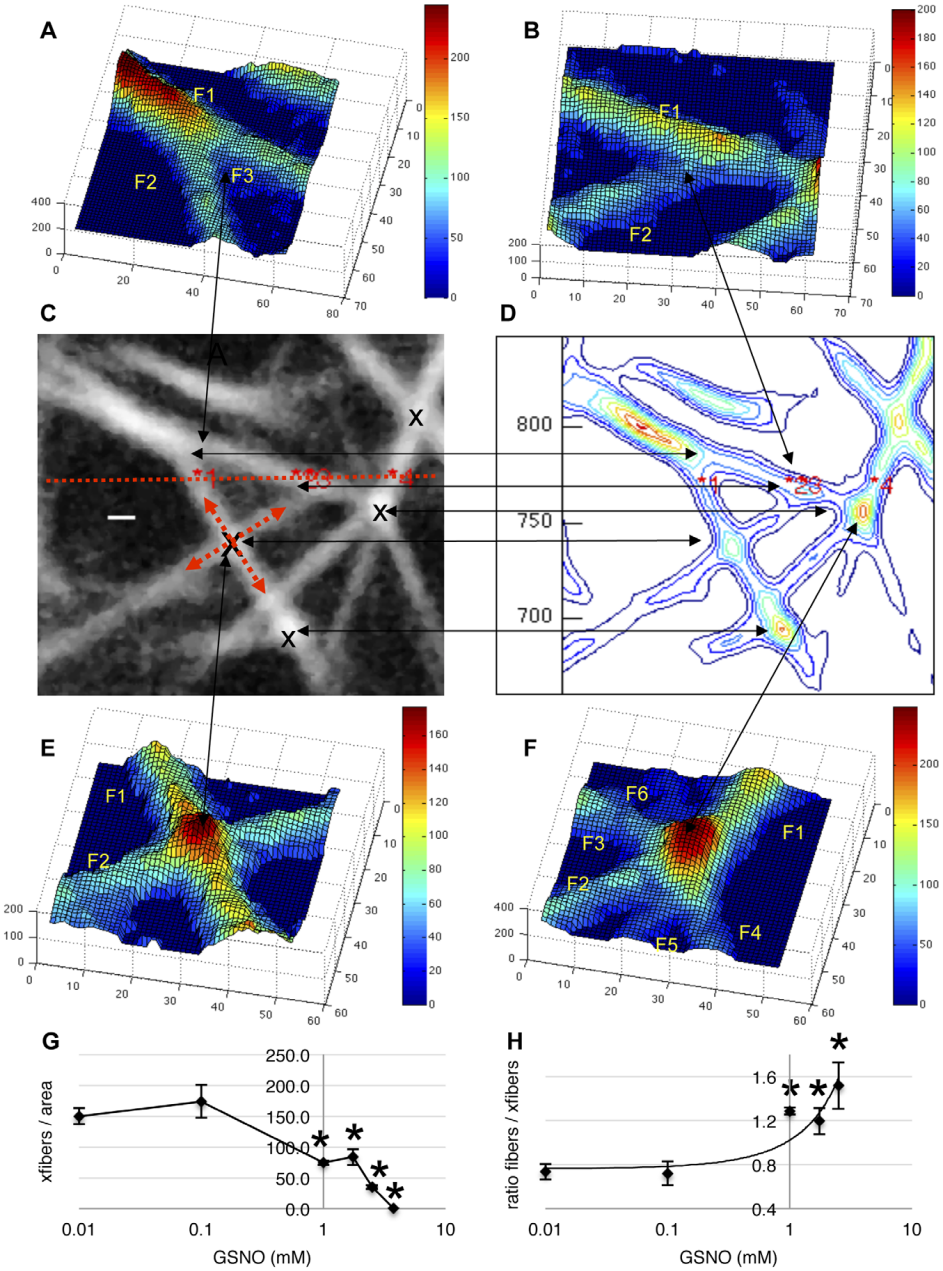
Crossing fibers are more sensitive than fiber density to GSNO. Images of native human fibrin clots were acquired using multiphoton microscopy and analyzed using custom designed computer software, as described in methods. Panels A,B,E,F show surface plots of fibrin fibers observed in the corresponding multiphoton image and contour figure, panels C,D respectively. Arrows connect the regions of interest. When two fibers cross at a point in space, there is a >40+/−15% increase in fluorescence intensity at the point of contact, that is proportional to fiber intensity. Panel A shows a trifunctional junction (F1 = F2+F3), panel B shows one fiber (F2) passing beneath another (F1), panel E shows two fibers (F1 and F2) crossing and panel F shows six fiber segments (or possibly three fibers) crossing at a point. As GSNO concentration increased, crossing fibers per area decreased, panel G. The ratio of fiber density to crossing fibers increased with increasing GSNO concentration, indicating the decrease in crossing fibers was greater than the decrease in fiber density, panel H. *p<0.05, compared to control. Area is 59×59 um^2^. Colour bars are image intensity. Scale bars are 700 nm.

We also calculated the fiber thin-thick ratio (TTR) based on the finding that fiber diameter was not normally distributed in control clots (Figure S2). The diameter data was fit to a bimodal distribution using the maximum likelihood estimation (MLE) method and the local minimum found to be 761.9 nm. This value was considered to be the threshold between thin and thick fibers. This was consistent with the report that plasma fibrin networks had a bimodal distribution of fiber thicknesses [35]. We found that fiber TTR decreased 74% (from 2.7±0.9 at 0.01 mM GSNO to 0.7±0.1) indicating fibers became thicker as GSNO concentration increased to 1 mM, before gradually becoming thinner again, TTR increased 85% (from 0.7±0.1 1.3±0.2) as GSNO increased to 2.5 mM. Taken together, as GSNO concentration increased, human fibrin fiber density decreased while fiber diameter increased to a maximum thickness before gradually decreasing as fibrin polymerization was inhibited. The change in fiber diameter was reflected by changes in the thin-to-thick fiber ratio and the appearance of abnormal fibrinogen cluster structural elements. These GSNO mediated changes in fiber diameter may be significant *in vivo*, as fiber diameter is a determinant of fibrinolysis, [36] suggesting that GSNO may function at an optimal concentration.

### Clot Void Volume is Relatively Resistant to Low Concentrations of GSNO

Fibrin network images contain spatial information that can be used to calculate the clot void area in a 2D image, and clot void volume over a stack of 2D images. Figure 3, panel A shows a series of control and GSNO binarized clot images, taken at 40,50,60 micron depths, and their respective projected images, for control (Figure 3C) and 2.5 mM GSNO (Figure 3B), respectively. Visual inspection revealed considerably more empty space in the GSNO treated clot than control. A plot of void volume against GSNO concentration showed that clot void volume remained relatively insensitive to GSNO below 1.75 mM (approx. 70%), indicating that spatial losses due to decreases in fiber density were offset by spatial increases in fiber diameter. However, as GSNO concentration increased beyond this point, there was a dramatic increase in void volume (from 72.7 to 85.2 to 100%) and projected void area (from 38.7 to 63.7 to 100%), Figure 3D, as both fiber density and fiber diameter decreased. These imaging results show that GSNO dramatically altered the porosity of human fibrin clots above a concentration threshold.

### GSNO Decreases Trifunctional Junctions, but has No Effect on the Fiber Density/Branch Point Ratio

During fibrin network formation, protofibrils are known to elongate and form trifunctional junctions. These branch points are characterized predominantly by three fibers intersecting at a point, where the width of one fiber equals the sum of widths of the other two fibers (F1 = F2+F3) [6], [7], [37] **.** Fiber branching gives rise to the complex nature of fibrin networks. In Figure 4 we show how trifunctional junctions can be readily identified in multiphoton images (Figure 4A) using a combination of contour maps (Figure 4B) and surface plots (Figure 4C) of the region of interest. As GSNO increased, branch point density per unit area was found to decrease from 48.7±8.3 to zero (branch points/area), Figure 4D. However, when compared to changes in fiber density (Figure 2D), the ratio of fiber density to branch point density was found to be insensitive to GSNO concentration. Our finding is similar to the report that the fiber density/branch point density ratio is a constant, and generally independent of clotting conditions [8]. (Note, we list branch point (trifunctional junctions) statistics as xbranches in clot “read out” images, Figure 2, panels A,B,C,G.)

### GSNO Decreased Crossing Fibers and Increased the Fiber Density/Crossing Fiber Ratio

In addition to trifunctional junctions, we also observed crossing fibers at a point in space within the network. These network structural elements were readily identified in contour images and surface plots, as shown in Figure 5, by high regions of intensity at the point of contact between fibers. During computer analysis, identified fibers were sampled along their respective lengths. If a local increase in fiber intensity was detected, then a search was made for a putative crossing fiber, which was similarly sampled along its length. If both fibers had proportional increases in intensity >40%, they were considered to be in contact; otherwise, one fiber was simply passing above or below the other, as shown in Figure 5B. As GSNO concentration increased, crossing fibers per unit area decreased, Figure 5G. When compared to changes in fiber density (Figure 2D), however, the ratio of fiber density to crossing fibers was found to increase from 0.7±0.1 to 1.5±0.2, p<0.05 with increasing GSNO concentration, Figure 5H, suggesting that crossing fibers were more sensitive to GSNO than fiber density. (Note, we list crossing fiber statistics as xlinks in clot “read out” images, Figure 2, panels A,B,C,G.)

### Abnormal Fibrinogen Polymerization and Clot Structures

**Figure 6:**
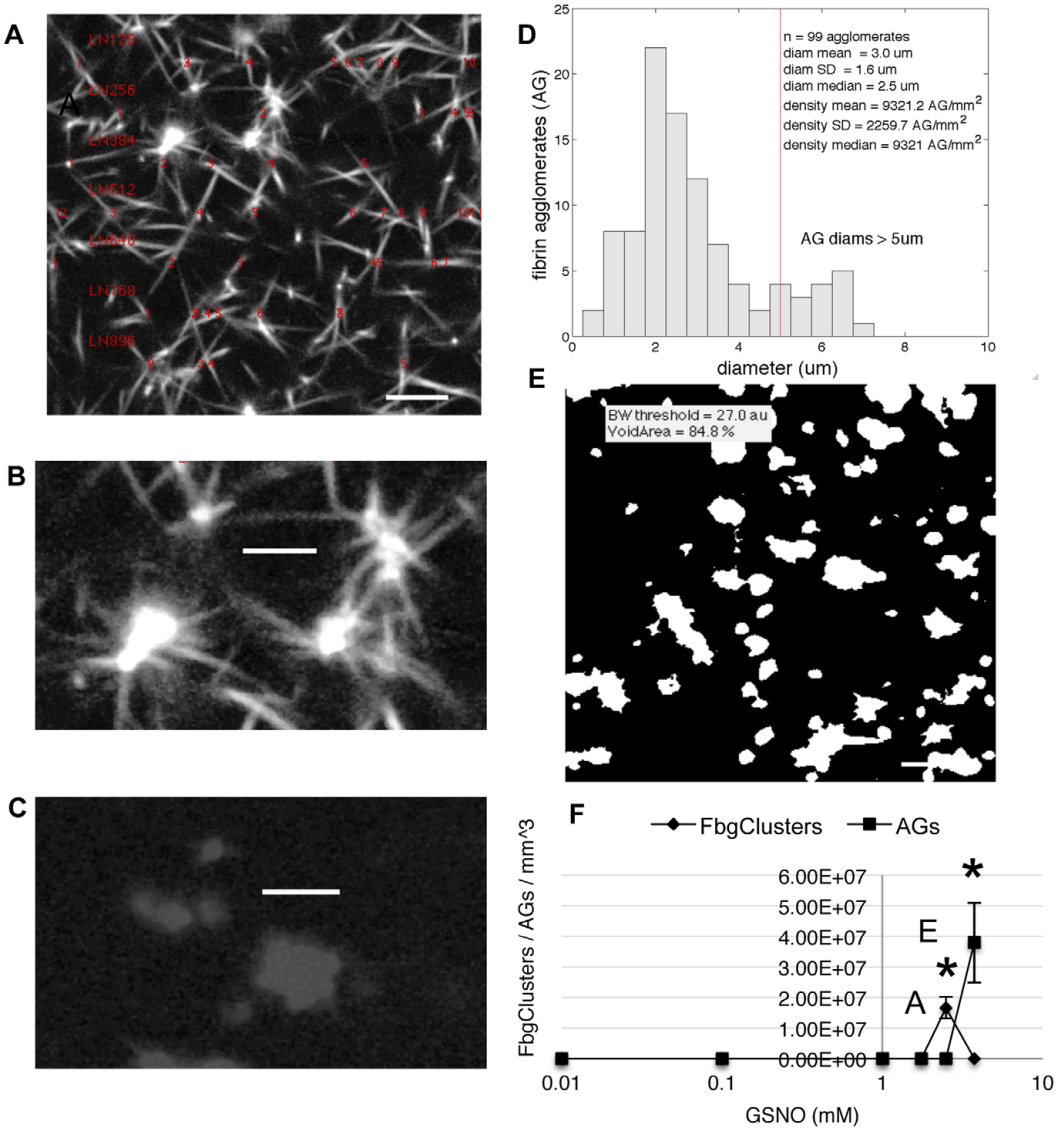
High concentrations of GSNO induced formation of abnormal fibrin clusters and fibrin agglomerates. Human fibrin clots were prepared and imaged as described in methods. When platelet-poor plasma was incubated with 2.5 mM GSNO, image analysis revealed the presence of abnormal fibrin clusters, characterized by high intensity cores with numerous protruding fibers, panels A and B. An unexpected finding was that 3.75 mM GSNO resulted in the formation of fibrin agglomerates, panel C. Panel D is a histogram of agglomerate long axis diameter. A projected binary image from 30,50,70 microns within the plasma sample, revealed the heterogeneous nature of the agglomerates, panel E. See data supplement figure 3 for additional images of agglomerates. Based on the GSNO dose-response curves, panel F, fibrin cluster formation preceded fibrin agglomerate formation. *p<0.05, compared to control. Scale bars for panels A,E are 8.5 um. Scale bars for panels B,C are 4.25 um.

As GSNO concentration increased to 2.5 mM, visual inspection of human fibrin clot images revealed the presence of abnormal network structures, referred to as fibrin clusters. Fibrin clusters were incorporated into the fibrin network and defined by an irregularly shaped high intensity core with >8–10 thin fibers protruding from the core periphery, Figure 6, panels A and B. And when plasma was treated with 3.75 mM GSNO, we discovered that fibrin fibers had been replaced by fibrin agglomerates. Fibrin agglomerates were suspended in plasma and had a range of irregular shapes and sizes (0.5–8 um) with some having thin blunted fibers protruding from the core, as seen in multiphoton and projected binary images, Figure 6, panel C and E, respectively, and Figure S3. Measurement along the agglomerate long axis found them to have a mean diameter of 2.8 um, with 11.5% having diameters greater than that of an average capillary (5–6 µm). Of note was that agglomerates were only detected at GSNO concentrations greater than that required to generate fibrin clusters, Figure 6, panel F.

## Discussion

### Summary

In this study, we used multiphoton microscopy to investigate the effects of S-nitrosoglutathione (GSNO) on native human fibrin clot architecture. We report for the first time that a series of structural changes occurred as fibrin network formation was progressively inhibited by increasing GSNO concentration. GSNO decreased fibrin density to the point of completely inhibiting fibrin network formation. Concomitantly, fiber diameter increased by 25% to a maximum diameter of 835 nm at 1 mM GSNO, after which fibers became thinner as GSNO was further increased. Image analysis showed that clot void volume (∼70%) was relatively insensitive to changes in GSNO over a lower concentration range (0.01–1.75 mM), as decreases in fibrin density were spatially offset by increases in fiber diameter. However, clot void volume rapidly increased as GSNO increased above this lower limit, as both fibrin density and fiber diameter decreased. This significant increase in clot void area coincided with the appearance of abnormal network structural elements, referred to here as “fibrin clusters”. These highly dense abnormal structures may signal the GSNO threshold for the onset of abnormal fibrin polymerization. Notwithstanding the obvious structural abnormalities, image analysis found no evidence of fiber movement, indicating the clot network remained stable [9]. This inherent stability was likely due, in part, to the finding that fiber branching was insensitive to GSNO concentration. Over the same concentration range, crossing fibers decreased, suggesting that clot stiffness was likely affected. All clots reached their respective gel points within 5 minutes of thrombin activation, except for human plasma treated with the highest GSNO tested. In this case, fibrin networks were completely inhibited and replaced, unexpectedly, by fibrin agglomerates.

### Changes in Fibrinogen Structure and Altered Fibrin Polymerization are Associated with GSNO Induced Changes in *Fibrin Clot Architecture*

Increasing evidence indicates that fibrinogen is susceptible to a variety of structural modifications [10], [16], [29], [30], [38], [39] that impact on its ability to polymerize and form fibrin networks. The mechanism whereby GSNO alters fibrinogen secondary structure is controversial. Akheter et al [29] reported that GSNO interacted with fibrinogen S-nitrosothiol binding domains in the aromatic rich αC-terminal region, increasing α-helical content and altering fibrinogen secondary structure. The effect was reversible, suggesting GSNO was an allosteric inhibitor. In contrast, Geer et al [30] proposed that GSNO alters fibrin polymerization by thiolating an exposed disulfide in the αC-region. While the mechanism remains uncertain, both studies found GSNO decreased the initial rate of polymerization. In the Geer study [30], this was related to increased lag time and decreased final turbidity. Here, we extended these biochemical findings by imaging and quantifying the nature and extent of the GSNO induced changes in fibrin network structure.

We found that GSNO decreased fibrin fiber density and fibrin branch point density while increasing fiber diameter, resulting in “coarse” fibrin networks, known to be more susceptible to fibrinolysis [36] and have weaker mechanical properties [8]. It is intriguing that GSNO maintained clot void volume, while altering fiber diameter, suggesting GSNO may modulate the remodeling or break down of fibrin networks over an optimal concentration range. Additionally, GSNO maintained the fiber density to branch point density ratio, an important feature of clot structure [37]; yet, it increased the fiber density to crossing fiber ratio, suggesting fiber interactions were more sensitive to GSNO than fiber branching. Taken together, GSNO functions as a small molecule modulator of fibrin network structure over a range of concentrations.

### Abnormal Fibrinogen Cluster Network Structural Elements

At low concentrations, GSNO decreased both fibrin fiber density and branch point density, while increasing fiber diameter. However, as GSNO concentration increased above 1.75 mM, we detected abnormal structural elements, characterized by a dense core of fibrin with numerous thin fibers protruding from the periphery. These abnormal fibrin clusters were incorporated into the fibrin network, as there was no evidence of fibrin movement within the image. Similar to our finding with GSNO, nitrated fibrinogen in the blood of cigarette smokers was found to generate fibrin networks with “clusters of fibrin” [16]. These “cluster” structures had >8 fibers crossing at a single point, and were related to the extent of fibrinogen nitration in the carboxy terminus of the fibrinogen β-chain (proximal region of the D domain). However, contrary to GSNO, nitrated fibrinogen increased the rate of fibrin polymerization, without affecting fibrin diameter. In another study, Marchi et al [38] reported that fibrin clots generated from human fibrinogen with a point deletion in the α-helical coiled-coil region (fibrinogen Caracus VI, deletion of Aα-Asn 80) resulted in fibrin networks with “some regions highly dense”. Consistent with GSNO, this dysfibrinogenemia decreased the rate of fibrin polymerization, but unlike GSNO there was no change in fiber diameter. Taken together, different structural modifications of fibrinogen (GSNO binding or thiolation within the αC region, nitration of tyrosine in the βC-terminal region or ASN80 deletion in the α-helical coiled-coil region), result in both increased and decreased rates of fibrin polymerization; yet, seemingly produce similarly abnormal fibrinogen cluster structures, suggesting that fibrinogen cluster structures result from a common, yet unknown, defect in the polymerization process.

### Fibrin Agglomerates

We report for the first time, that high concentrations of GSNO completely inhibit fibrin network formation, while generating a heterogeneous population of fibrin agglomerates. These agglomerates were heterogeneous in both size (0.5–8 µm) and shape (spherical to irregular). Image analysis revealed that some of the agglomerates shared structural features with fibrin clusters (blunted protruding fibers form a central core), suggesting the latter may be intermediate forms of the former. It is reasonable to assume fibrin agglomerates consist of short protofibrils and oligomers that have failed to elongate and branch, but have retained the ability to laterally aggregate, or agglomerate. The role these agglomerates may play *in vivo* is unknown; however, if not quickly recycled into platelets or cleared from the circulation, they may increase the risk of microvascular complications given that a large number of agglomerates exceed the luminal diameter of capillary beds (∼5–6 um) and could thereby block capillary blood flow.

In contrast to fibrin agglomerates generated during fibrin polymerization in the presence of GSNO, which remained in suspension, Sakharov et al. [32] reported that fibrin agglomerates (2–4 um) formed within a 5–8 micron superficial layer during fibrinolysis, eventually dissolved. Accordingly, fibrin agglomerates seemingly play a beneficial role in clearing fibrin fragments during fibrinolysis, or a potentially deleterious role if generated under coagulation conditions and not removed from the circulation. This may be of significance in the design of drug-eluting stents to prevent restenosis, where GSNO is combined with a polymeric coating, and has the property of inhibiting platelet aggregation with an EC50 of 5.0 mM [28]. Our results suggest this concentration of GSNO could induce local formation of fibrin agglomerates that may enter the circulation and potentially plug the downstream microcirculation.

### RBC-GSNO Theoretical Mechanism for Thrombus Remodeling

While nitrosative stress can lead to increased circulating levels of S-nitrosothiols [17] the relatively high *in vitro* concentrations of GSNO required to first alter fibrin network architecture and then inhibit fibrin formation suggests that local sources of GSNO would likely be involved *in vivo*. Possible local sources of GSNO within blood clots are red blood cells, which release S-nitrosothiols in response to hypoxic gradients [19], [40], [41] platelet derived nitric oxide [42] and S-nitrosylated-albumin [43] which could both transnitrosate [44] plasma glutathione forming GSNO. Under the appropriate hypoxic conditions, which may exist for example in an intraluminal thrombus [45], it is possible that trapped erythrocytes within a fibrin network modulate on-going fibrin polymerization and remodel clot architecture [46] by releasing GSNO into the local clot environment, where it is free to diffuse within the clot and interact with fibrinogen or S-nitrosylate the free sulfhyryl group in factor XIIIa [31]. While the theory that RBC GSNO modulates fibrin architecture is intriguing, and somewhat complementary to the finding that RBCs carrying plasminogen activators modulate clot lysis [47], more research is required to determine if such a mechanism occurs in vivo.

### Conclusion

The picture that emerges from this study is that GSNO dramatically alters human fibrin clot architecture in a dose-dependent manner, acting as a small molecule modulator of fibrin clot architecture. While many structural modifications of fibrinogen are associated with fine fibrin clot structures that are stiffer and resistant to clot lysis, GSNO produces coarse clot networks with decreased fibrin density and increased fiber diameter. Such clots are known to be more susceptible to clot lysis [36]; though, further research is required to determine the effects of GSNO on fibrinolysis. Our image analysis clearly shows that during fibrin polymerization, fibrin network architecture is sensitive to elevated GSNO concentration, where excessive GSNO leads to abnormal fibrin networks and even the generation of fibrin agglomerates. We conclude that GSNO may attenuate the risk of thrombosis and facilitate thrombus remodeling at low levels, but induce fibrin polymerization disorders at high levels.

## Supporting Information

Figure S1 **Computer analysis of fibrin clot networks.** Fibrin clot images were acquired using multiphoton microscopy and analyzed using in-house custom designed software. A series of horizontal test lines along the image y-axis (at LN171,LN341,LN512,LN682,LN853) were generated to sample fluorescence intensity and determine fiber density. The corresponding intensity profiles are shown in panel B, where the various intensity peaks correspond to fluorescent fibers. The software then numbered and mapped all peaks in the intensity profile back into the clot image and calculated fiber density as the number of fibers per 100 um test line. The software program is interactive and allowed the operator to verify the identity of each fiber and edit false positives or false negative fibers, if necessary, using the graphical user interface shown in panel C. The four panels on the left, from top to bottom, are two subimages of the test line being evaluated (LN512), showing the test line in red and the numbered fibers. The bottom two panels are the intensity profile and first derivative plot of the intensity profile, respectively. More than one fiber was found to intersect at a branch point, as shown in the dashed circle area. Next, the diameter of each fiber was determined automatically. The right side of panel C shows a subimage of a fiber and the position where the diameter measurement was made. The solid circle corresponds to the fiber measurement. Automatic diameter measurements were validated against manual diameter measurements, panel D, and found to be in good agreement, panel E, p<0.001. Scale bar is 8.5 um.
(PDF)

Figure S2 **Effect of GSNO on fiber fluorescence and fiber diameter.** Human fibrin clots were prepared and imaged as described in methods. Panels A-E show binarized (BW theshold  = 27au) multiphoton images of fibrin clots incubated with GSNO (0,1,1.7,2.5,3.75 mM, respectively). Panels F,I,L show fiber diameter information, where each panel consists of three plots (top to bottom), diameter histogram, normality plot and normal or bimodal fit to the distribution; panels G,J,M show fiber fluorescence distributions; panels H,K,N show plots of fiber diameter against fiber fluorescence, for clots prepared with 0,1,2.5 mM GSNO, respectively. The diameter distribution in control clots was not normally distributed. A bimodal fit to the data found a value of 761.9 nm to be the threshold between thin and thick fibers. Solid lines are the mean value for the distribution. Dashed lines show the control threshold value. Fiber diameter was related to fiber fluorescence as expected, p<0.05 for all GSNO concentrations tested. Scale bars are 8.5 um.
(PDF)

Figure S3 **Fibrin agglomerates.** When fibrinogen was incubated with 3.75 mM GSNO for 10 minutes at 37°C and then treated with thrombin 1(U/ml), only fibrin agglomerates were observed in the plasma sample. Panels A-L show agglomerates were heterogeneous in both size and shape. Their size ranged from 0.5–8 microns along the major agglomerate axis, while their shape ranged from small spherical agglomerates (A,B,C,D) to larger irregular agglomerates (G,H,I,L) with sizes up to 8 microns along the major axis. Interestingly, several agglomerates (H,J,K) shared structural similarities with fibrin clusters; although, their protruding fibers were blunted compared to fibrin clusters observed within clot network. Scale bars are 4.25 microns.
(PDF)
