# Supplementary figures and images for: S-Nitrosoglutathione Acts as a Small Molecule Modulator of Human Fibrin Clot Architecture
Source: PLoS One. 2012 Aug 20;7(8):e43660. doi: 10.1371/journal.pone.0043660 (PMC3423378; doi:10.1371/journal.pone.0043660)

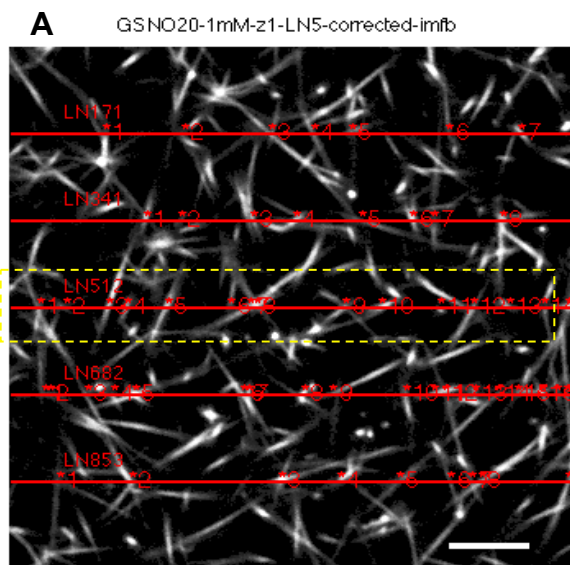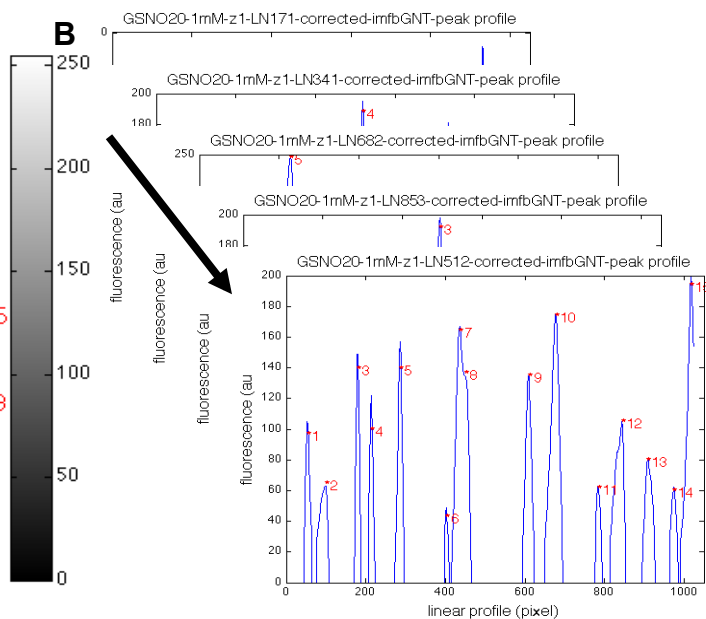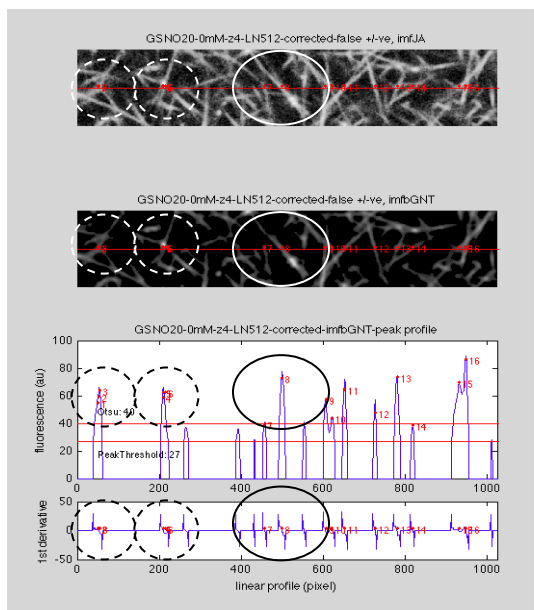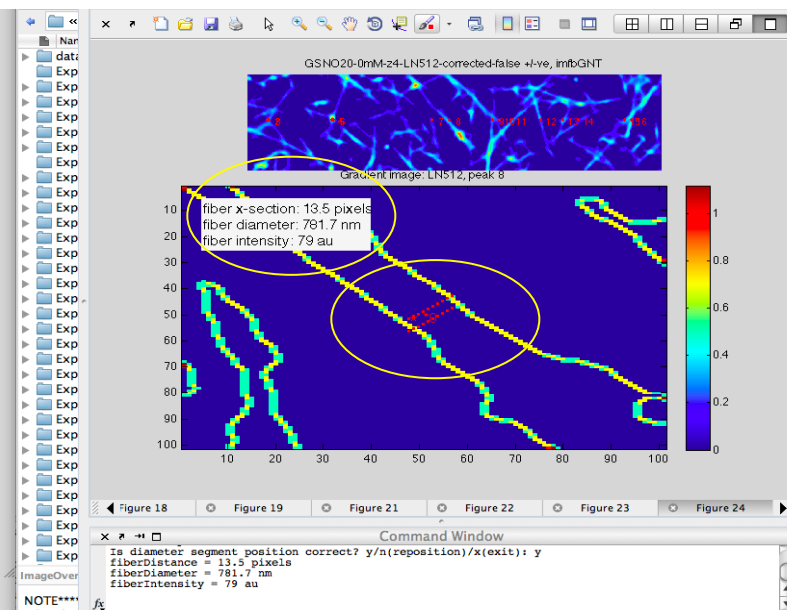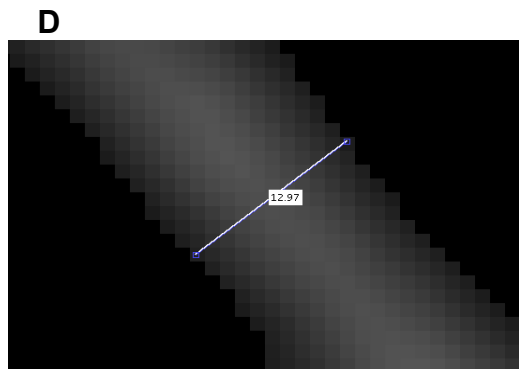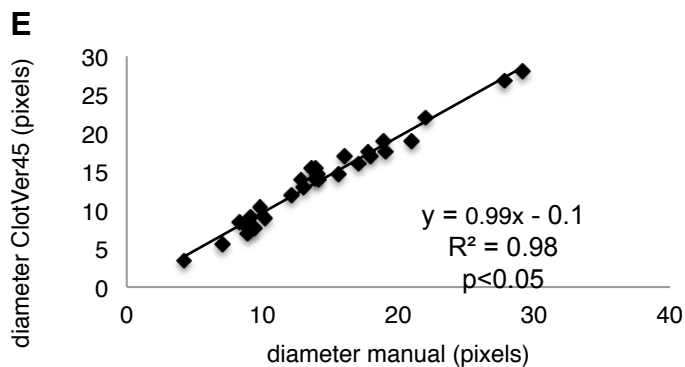

Supplement: Figure S1 — Computer analysis of fibrin clot networks. Fibrin clot images were acquired using multiphoton microscopy and analyzed using in-house custom designed software. A series of horizontal test lines along the image y-axis (at LN171,LN341,LN512,LN682,LN853) were generated to sample fluorescence intensity and determine fiber density. The corresponding intensity profiles are shown in panel B, where the various intensity peaks correspond to fluorescent fibers. The software then numbered and mapped all peaks in the intensity profile back into the clot image and calculated fiber density as the number of fibers per 100 um test line. The software program is interactive and allowed the operator to verify the identity of each fiber and edit false positives or false negative fibers, if necessary, using the graphical user interface shown in panel C. The four panels on the left, from top to bottom, are two subimages of the test line being evaluated (LN512), showing the test line in red and the numbered fibers. The bottom two panels are the intensity profile and first derivative plot of the intensity profile, respectively. More than one fiber was found to intersect at a branch point, as shown in the dashed circle area. Next, the diameter of each fiber was determined automatically. The right side of panel C shows a subimage of a fiber and the position where the diameter measurement was made. The solid circle corresponds to the fiber measurement. Automatic diameter measurements were validated against manual diameter measurements, panel D, and found to be in good agreement, panel E, p<0.001. Scale bar is 8.5 um. (PDF) [file pone.0043660.s001.pdf]

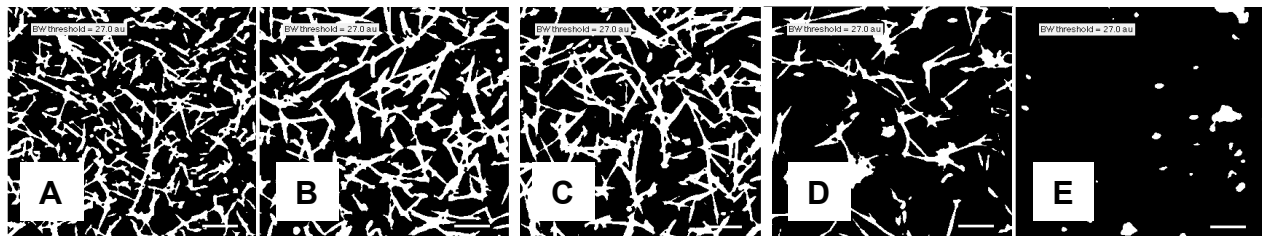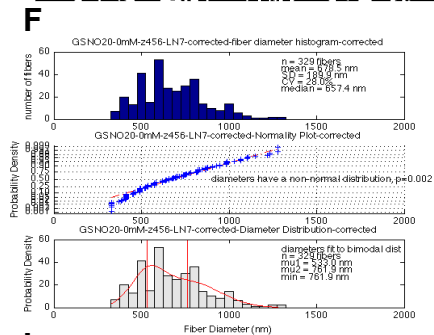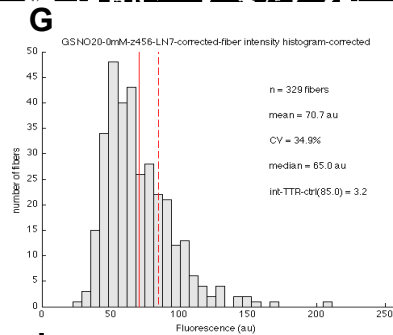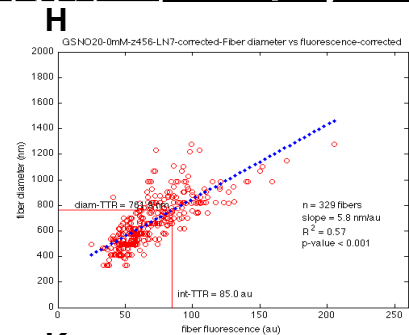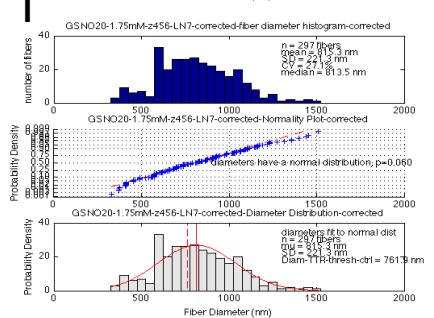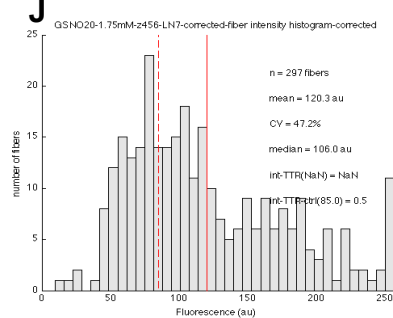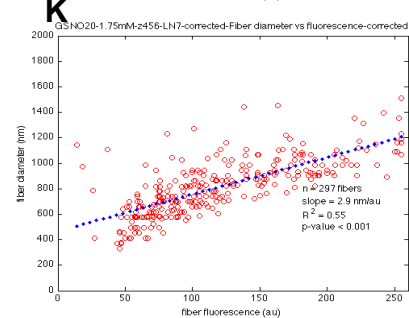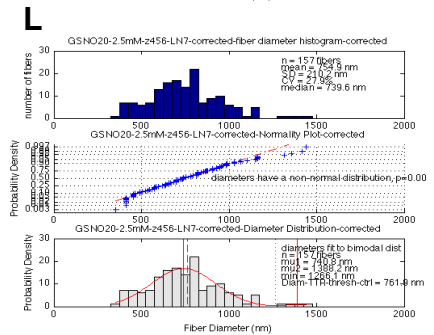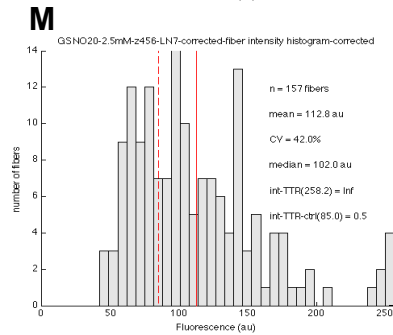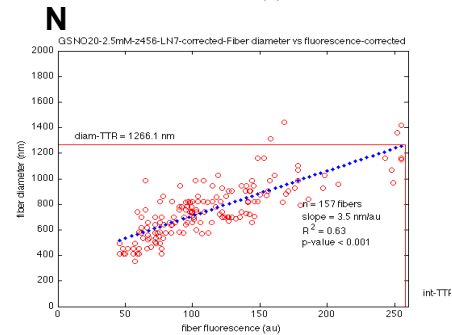

Supplement: Figure S2 — Effect of GSNO on fiber fluorescence and fiber diameter. Human fibrin clots were prepared and imaged as described in methods. Panels A-E show binarized (BW theshold = 27au) multiphoton images of fibrin clots incubated with GSNO (0,1,1.7,2.5,3.75 mM, respectively). Panels F,I,L show fiber diameter information, where each panel consists of three plots (top to bottom), diameter histogram, normality plot and normal or bimodal fit to the distribution; panels G,J,M show fiber fluorescence distributions; panels H,K,N show plots of fiber diameter against fiber fluorescence, for clots prepared with 0,1,2.5 mM GSNO, respectively. The diameter distribution in control clots was not normally distributed. A bimodal fit to the data found a value of 761.9 nm to be the threshold between thin and thick fibers. Solid lines are the mean value for the distribution. Dashed lines show the control threshold value. Fiber diameter was related to fiber fluorescence as expected, p<0.05 for all GSNO concentrations tested. Scale bars are 8.5 um. (PDF) [file pone.0043660.s002.pdf]

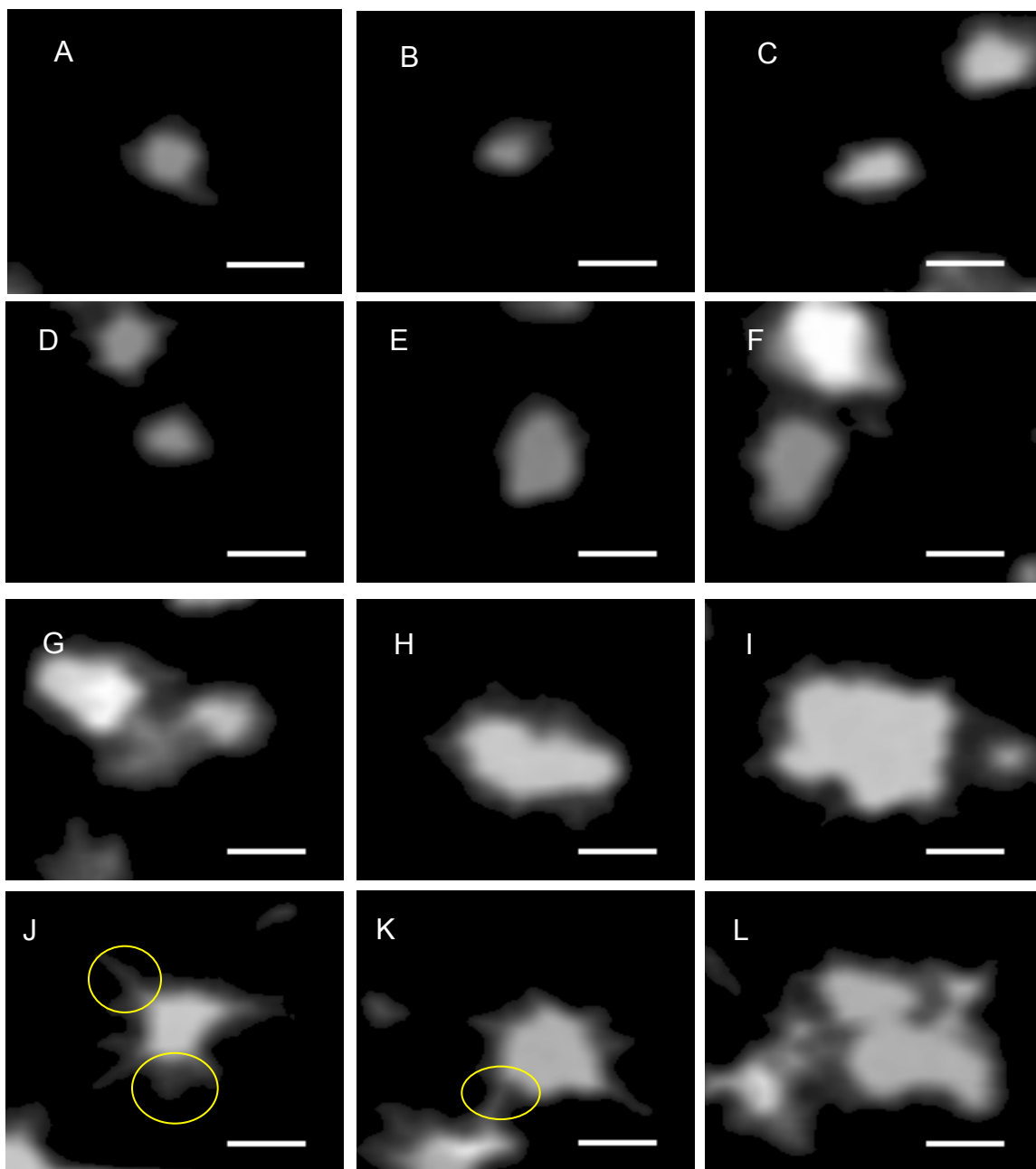

Supplement: Figure S3 — Fibrin agglomerates. When fibrinogen was incubated with 3.75 mM GSNO for 10 minutes at 37°C and then treated with thrombin 1(U/ml), only fibrin agglomerates were observed in the plasma sample. Panels A-L show agglomerates were heterogeneous in both size and shape. Their size ranged from 0.5–8 microns along the major agglomerate axis, while their shape ranged from small spherical agglomerates (A,B,C,D) to larger irregular agglomerates (G,H,I,L) with sizes up to 8 microns along the major axis. Interestingly, several agglomerates (H,J,K) shared structural similarities with fibrin clusters; although, their protruding fibers were blunted compared to fibrin clusters observed within clot network. Scale bars are 4.25 microns. (PDF) [file pone.0043660.s003.pdf]
